# Phytoplasma DNA Enrichment from Sugarcane White Leaves for Shotgun Sequencing Improvement

**DOI:** 10.3390/plants13213006

**Published:** 2024-10-28

**Authors:** Karan Lohmaneeratana, Gabriel Gutiérrez, Arinthip Thamchaipenet, Ralf Erik Wellinger

**Affiliations:** 1 Department of Genetics, Faculty of Science, Kasetsart University, Bangkok 10900, Thailand; karan.l@ku.th; 2 Centro Andaluz de Biología Molecular y Medicina Regenerativa, Universidad de Sevilla, 41092 Sevilla, Spain; 3 Departamento de Genética, Universidad de Sevilla, 41012 Sevilla, Spain; ggpozo@us.es; 4 Omics Center for Agriculture, Bioresource, Food and Health Kasetsart University (OmiKU), Bangkok 10900, Thailand

**Keywords:** phytoplasma, DNA size selection, microbiome enrichment, metagenome, MAG

## Abstract

Sugarcane white leaf (SCWL) disease, caused by *Candidatus* Phytoplasma sacchari, poses a significant threat to sugarcane cultivation. An obligate parasite, phytoplasma is difficult to culture in laboratory conditions, making the isolation of its DNA from the massive amount of plant host DNA extremely challenging. Yet, the appropriate amount and quality of plant microbiome-derived DNA are key for high-quality DNA sequencing data. Here, a simple, cost-effective, alternative method for DNA isolation was applied using a guanidine-HCl-hydroxylated silica (GuHCl-Silica)-based method and microbiome DNA enrichment based on size-selective low-molecular-weight (LMW) DNA by PEG/NaCl precipitation. qPCR analysis revealed a significant enrichment of phytoplasma DNA in the LMW fraction. Additionally, the NEBNext Microbiome DNA enrichment kit was utilized to further enrich microbial DNA, demonstrating a remarkable increase in the relative abundance of phytoplasma DNA to host DNA. Shotgun sequencing of the isolated DNA gave high-quality data on the metagenome assembly genome (MAG) of *Ca.* Phytoplasma sacchari SCWL with completeness at 95.85 and 215× coverage. The results indicate that this combined approach of PEG/NaCl size selection and microbiome enrichment is effective for obtaining high-quality genomic data from phytoplasma, surpassing previous methods in efficiency and resource utilization. This low-cost method not only enhances the recovery of microbiome DNA from plant hosts but also provides a robust framework for studying plant pathogens in complex plant models.

## 1. Introduction

Plant-associated microorganisms have important pros (beneficial roles) and cons (pathogenic roles) in agricultural crop growth and health. Sugarcane is an economically important plant that suffers from various pathogens. Among pathogenic bacteria, phytoplasma is the most destructive pathogen, causing sugarcane diseases including sugarcane grassy shoot (SCGS) and sugarcane white leaf (SCWL), which have severe consequences for crop yield and quality [1]. SCWL disease leads to symptomatic changes; sugarcane leaves become thin and narrow, turning yellow to completely chlorotic. The treatment or protection of plants against phytoplasma is rather difficult and unfriendly to the environment [2].

The characterization and monitoring of plant pathogens over time are essential for controlling their spread. This process often requires DNA extraction and separation from plant tissue collection and subsequent plant and microbial DNA analysis. Hybrid sugarcane cultivars contain 110–120 chromosomes, comprising a genome of up to 10 Gb [3]; effective DNA isolation and storage methods are crucial in maintaining the integrity of both plant and microbial DNA. In addition to the nuclear genome, sugarcane tissues contain DNA from smaller genomes, such as chloroplasts (141 kb), mitochondria (between 145 and 301 kb), and microbes [4,5]. However, suboptimal conditions can lead to DNA fragmentation, making it less suitable for downstream applications such as whole-genome sequencing (WGS) [6]. Thus, the separation of enriched microbial DNA by size-selective DNA separation, as well as the verification that this DNA is suitable for shotgun genome sequencing, were investigated.

Recent advances in next-generation sequencing technology have made it possible to obtain complete or nearly complete genomes of phytoplasma. The whole genome sequencing of *Ca.* Phytoplasma sacchari SCGS and SCWL1 isolated from India and China, respectively [5,7], has provided new insights into the genetic makeup of these pathogens and their potential mechanism of pathogenicity. The SCGS and SCWL1 strains were sequenced using Illumina and Oxford Nanopore technology [5,7]. These techniques were essential in addressing, with high resolution and low cost, the challenges of the small amount of phytoplasma DNA within the sugarcane tissue and the overwhelming presence of the plant’s own DNA [8]. Since phytoplasma DNA is often much less abundant compared with the host plant’s DNA, it can be difficult to isolate and analyze. These techniques helped with effectively separating and enriching the phytoplasma DNA, making it possible to study it despite these challenges.

In response to these challenges, in this study, a novel DNA isolation protocol was developed to avoid the use of hazardous chloroform and to enable the size selection of DNA fragments, facilitating the enrichment of microbial DNA from sugarcane tissues. By combining guanidine hydrochloride (GuHCl) and hydroxylated silica beads for DNA isolation together with selective polyethylene glycol (PEG) precipitation, high-molecular-weight (HMW) and low-molecular-weight (LMW) DNA were effectively separated. Furthermore, by integrating this approach with the NEBNext Microbiome DNA enrichment kit, the recovery and quality of phytoplasma genomes were improved. This method not only preserves the integrity of microbial DNA during extraction but also ensures its suitability for downstream applications, such as shotgun sequencing, offering a cost-effective and efficient tool for the study of plant-associated pathogens.

## 2. Results

### 2.1. Separation of High- and Low-Molecular-Weight DNA by PEG/NaCl Precipitation

**Figure 1:**
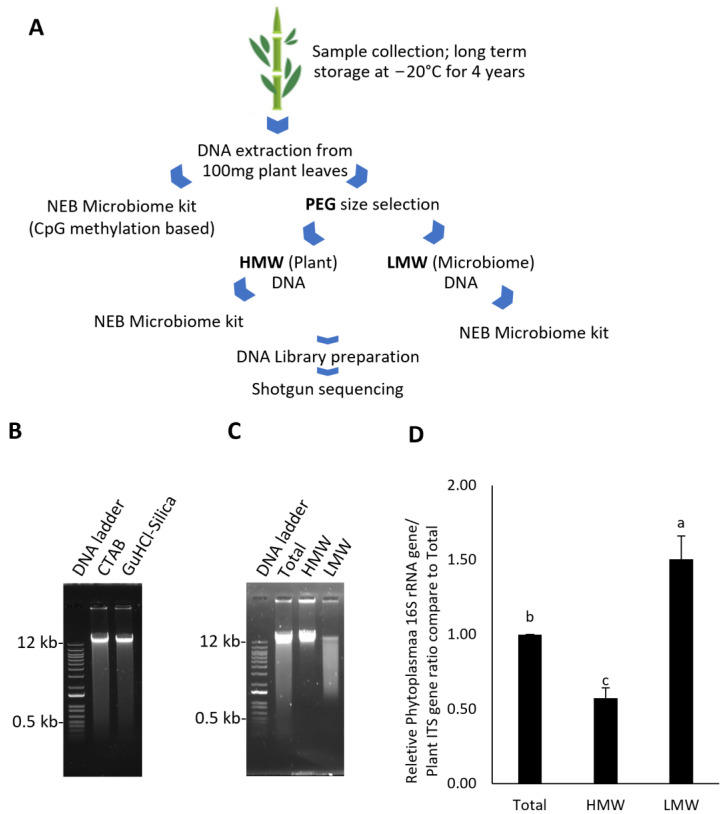
DNA size selection by the PEG-based enrichment protocol. (**A**) Schematic outline of the HMW and LMW DNA enrichment. (**B**) Comparison of total DNA derived from CTAB and GuHCl-silica-based DNA isolation methods. (**C**) Comparison of total, HMW, and LMW DNA isolations. (**D**) qPCR analysis of 16S rDNA of phytoplasma versus ITS of sugarcane from total, HMW, and LMW DNAs. Different letters indicate significant differences.

In this work, an alternative DNA isolation protocol is proposed to avoid the use of chloroform and enable the size selection of DNA. Moreover, PEG/NaCl was employed for the size-selective precipitation of HMW and LMW DNA (outlined in Figure 1A and Figure S1). When comparing the quality of total DNA derived from GuHCl-Silica and cetyltrimethylammonium bromide (CTAB) using DNA isolation methods, there was no visible difference between the migration pattern of DNA isolated by both protocols (Figure 1B). DNA extraction protocols are practically expected to affect DNA integrity, and thus, fast-migrating DNA (smear) corresponding to the presence of broken DNA fragments was usually observed. This result suggests that the integrity of most cellular DNA was barely affected by the DNA extraction methods.

The GuHCl-Silica-extracted DNA was then separated into HMW and LMW DNA by precipitation with PEG/NaCl solution. HMW DNA was precipitated first, which was followed by a second precipitation process to retrieve the LMW DNA from the supernatant. The results indicate that selective PEG/NaCl precipitation assisted the enrichment of HMW and LMW GuHCl-Silica-extracted DNA and separated both fractions by sizing DNA larger than 12 kb (HMW) from 0.5–12 kb (LMW) (Figure 1C).

The relative ratio of phytoplasma DNA to sugarcane genomic DNA of total, HMW, and LMW DNA fractions was assessed through qPCR analysis of 16S ribosomal DNA (rDNA) of the phytoplasma and internal transcribed spacer (ITS) region of sugarcane. The relative amount of phytoplasma 16S rDNA/sugarcane gDNA decreased by 0.57-fold in the HMW fraction and increased by 1.5-fold in the LMW fraction when compared with the total DNA fraction (Figure 1D). This result indicates that selective PEG/NaCl precipitation allows for the separation of host and pathogenic DNA according to different genome sizes, enriching the microbial DNA.

### 2.2. Improvement of Microbial DNA Quality by Microbiome Enrichment Kit

**Figure 2:**
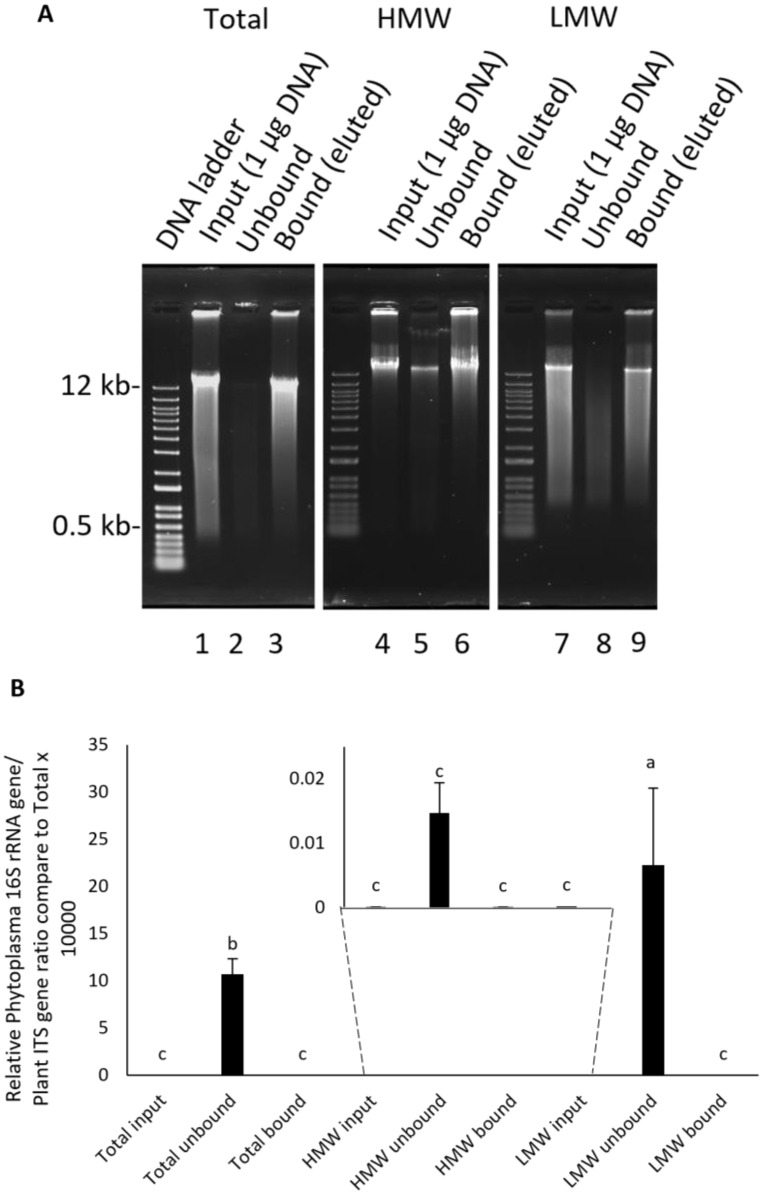
Comparison of CpG-methylated DNA (bound) and non-CpG DNA (unbound) from total, high (HMW), and low molecular weight (LMW) DNA fractions purified by NEBNext Microbiome DNA Enrichment kit. (**A**) Lanes 1, 4, and 7 are input DNAs (non-purified); lanes 2, 5, and 8 are unbound DNA; and lanes 3, 6, and 9 are bound DNA obtained from total, HMW, and LMW fractions, respectively. (**B**) qPCR analysis 16S rDNA of phytoplasma versus ITS of sugarcane. Different letters indicate significant differences.

In this work, a commercially available NEBNext Microbiome DNA enrichment kit (New England Biolabs, Ipswich, MA, USA) was used to separate microbial DNA from sugarcane gDNA in total, HMW, and LMW fractions. The CpG methylated sugarcane DNA was bound to MBD2-Fc-bound paramagnetic beads, while the non-CpG microbial DNA was unbound and subsequently collected in the supernatant portion. Strikingly, DNA sizes ranging from 0.5 to 12 kb were mostly enriched from the LMW fraction (Figure 2A, lane 8), while the least enrichment was observed in the total and HMW DNA fractions (Figure 2A, lanes 2 and 5, respectively). The relative amounts of phytoplasma 16S rDNA to the ITS of sugarcane of the enriched (unbound) total (en-Total), LMW (en-LMW), and HMW (en-HMW) DNA fractions increased 10^−6^, 2.2 × 10^−6^, 10^2^-fold, respectively, when compared to the total DNA input fraction (non-purified) (Figure 2B). These results clearly demonstrated the recovery improvement of phytoplasma DNA enrichment by a combination of PEG/NaCl-dependent size selection with the exclusion of CpG-methylated DNA.

### 2.3. Metagenome Shotgun Sequencing Analysis

The relevant goals of this study included the development of a simple, non-toxic DNA isolation protocol from SCWL sugarcane that allows the selective enrichment of microbial DNA by PEG/NaCl precipitation and to assess the quality of SCWL phytoplasma genomic DNA by shotgun sequencing. Therefore, 75-bp and 150-bp paired-end shotgun sequencing was performed. Given that the ratio of phytoplasma 16S rDNA was mostly enriched in the LMW fraction (Figure 2B), only these sequencing data were analyzed. The percentage mapping to the sugarcane reference genome was highest in non-enriched LMW DNA (LMWWO) in both 75-bp (LMWWO_75) and 150-bp (LMWWO_150) paired-end data, which was followed by enriched HMW (en-HMW), 75-bp paired-end enriched LMW (en-LMW_75), 150-bp paired-end enriched LMW (en-LMW_150), and enriched total (en-Total) DNA data (Table S1).

**Figure 3:**
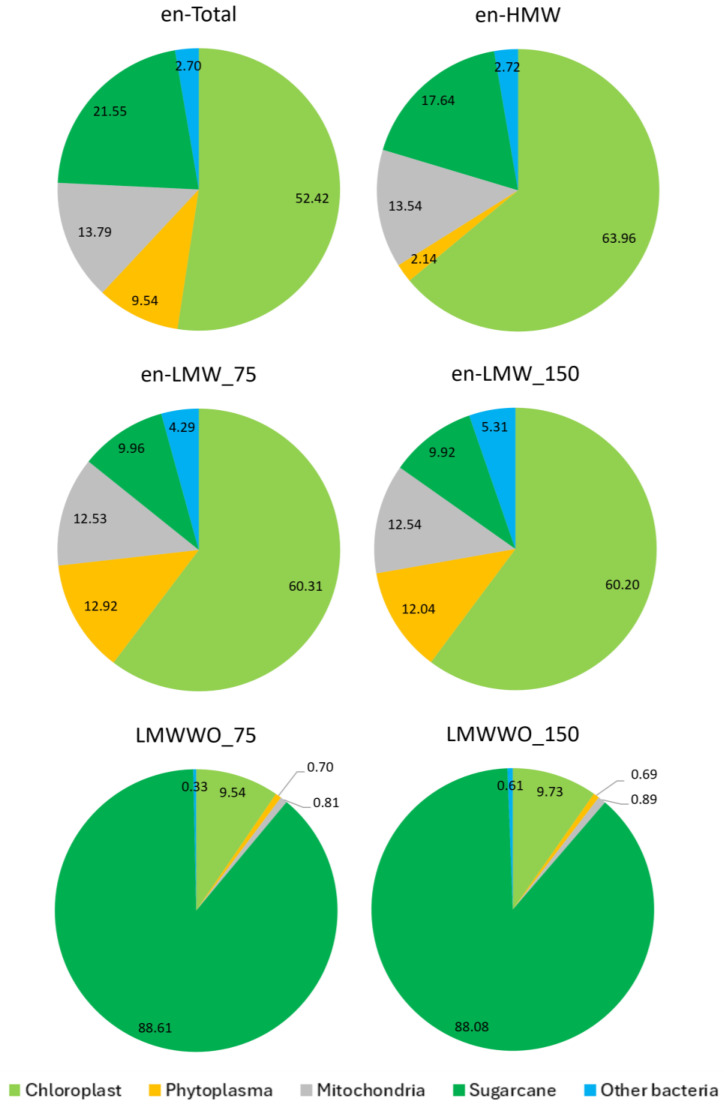
Percentage of chloroplast, phytoplasma, mitochondria, sugarcane, and other bacterial DNAs based on Kraken taxonomy classification of the shotgun metagenome data of enriched total (en-Total), enriched HMW (en-HMW), 75-bp paired-end enriched LMW (en-LMW_75), 150-bp paired-end enriched LMW (en-LMW_150), 75-bp paired-end non-enriched LMW (LMWWO_75), and 150-bp paired-end non-enriched LMW (LMWWO_150).

Clean reads were subjected to Kraken taxonomy classification. The relative abundances of reads of sugarcane, chloroplast, mitochondria, phytoplasma, and other bacterial DNAs were obtained (Figure 3). As expected, sugarcane DNA was highly represented in the LMWWO (88%), while only 10–20% were found in all enriched fractions. After the enrichment procedure, the abundance of phytoplasma and other bacteria was highly enriched in en-LMW data at 12% and 5%, respectively, when compared to those of en-HMW (about 2%) (Figure 3). However, chloroplast and mitochondrial DNAs constituted up to 50–60% (about 13%), respectively, of all enriched fractions. Kraken taxonomy classified the microbiome data indicating the presence of bacteria belonging to Acholeplasmatales (phytoplasma), Actinobacteria, Alphaproteobacteria, Anaerolineae, Bacillales, Bacteroidales, Bukholderiales, Desulfobactereota, Endomicrobiales, Lactobacillales, Mycoplasmatales, and Oscillospirales. Among these, Acholeplasmatales, represented a significant portion, comprising 9.80% of the en-Total and exhibiting high levels of representation across all DNA fractions, particularly 12.97% in the en-LMW_75 and 12.70% in en-LMW_150 fraction. Other prominent groups included the Endomicrobiaceae with 0.38% of the en-Total and 0.58% in en-LMW_75. Mycoplasmatales represented 1.31% and 1.03% in en-LMW_75 and 150, respectively. Other bacterial groups, including Actinobacteria, Bacillales, Lactobacillales, and Oscillospirales were detected at lower percentages with varying degrees of representation across the different DNA fractions. This detailed distribution across different DNA fractions highlights the significant enrichment of certain bacterial groups, particularly Acholeplasmatales, which shows a notably higher presence in the LMW fractions, suggesting an elevated abundance of phytoplasma-related organisms in these samples.

### 2.4. Metagenome-Assembled Genomes (MAGs) of Ca. Phytoplasma Sacchari SCWL

By analyzing the quality and genome coverage of phytoplasma (data analysis is outlined in Figure S2), there is a striking difference in the reconstruction of the phytoplasma genome size between en-Total, en-HMW, and en-LMW shotgun sequencing data (Table 1). Only about 49% of the phytoplasma genome was completely assembled as MAG from the en-Total despite it having a coverage of 32×. The quality of the shotgun sequencing data of the en-HMW was not sufficient to meet the binning service (BS) criteria required for MAG analysis. Outstandingly, both en-LMW_75 and en-LMW_150 gave the most complete phytoplasma genome assembly at about 98%, although the coverage of en-LMW_75 (29×) was much lower than that of en-LMW_150 (64×) (Table 1). Evidently, this improvement in genome assembly agrees with the double enrichment procedures applied (Table 1). In this case, the microbial DNA in the LMW sample enriched by selective PEG/NaCl precipitation alone (without further enrichment by the commercial microbiome DNA enrichment kit) is sufficient for the shotgun sequencing analysis of the phytoplasma genome. Regardless of the 75- or 150-bp paired-end sequencing data, achieving 98% of the phytoplasma genome coverage required 40% of the submitted reads to be consigned to binning. In addition, the MAG analysis of shotgun sequencing data from the non-enrichment (LMWWO_150) resulted in 74% genome assembly with a coverage of 18× (Table 1).

The genome sequence of *Ca*. Phytoplasma sacchari SCWL obtained in this work was generated by co-assembly sequences from all fractions (en-Total, en-HMW, en-LMW_75, en-LMW_150, and LMWWO_150; Table 2). This comprehensive co-assembly approach resulted in a high-quality genome sequence that is comparable to those available in existing databases. The process successfully enhanced the MAG of *Ca.* Phytoplasma sacchari SCWL at 95.85% completeness with 215× coverage by reducing the number of contigs to 45 and resulting in a complete genome size of 533,100 bp (Table 2).

## 3. Discussion

Apart from nuclear, chloroplast, and mitochondrial genomes, plant tissue contains DNAs of microbial or viral origin [9]. The difference in sizes amongst these genomes makes them suitable for genome size-dependent separation approaches. However, prior to size separation, genomes must be purified from the plant tissue. There are several protocols to separate DNA, which have been established to isolate pure and intact whole genomic DNA from plant tissues [10,11]. DNA from SCWL sugarcane was generally isolated by a ‘classical’ protocol including CTAB and chloroform [12]. However, in this study, a non-toxic, efficient DNA isolation protocol using the chaotropic agent, guanidine hydrochloride (GuHCl), was employed for membrane lysis and protein denaturation to minimize mechanical shear forces during the lysis step, which helps to preserve the integrity of DNA, making it suitable for applications requiring long DNA fragments [13]. A chloroform mixture is commonly added to lyse or homogenize biological material and separate unwanted proteins and cellular debris into the organic phase by centrifugation, allowing the DNA to remain in the aqueous phase for easy isolation and analysis. However, due to the significant health risk posed by hazardous organic solvents [14], this process requires careful risk assessment and proper handling [15]. In this study, instead of chloroform, hydroxylated silica beads coated with citric acid were applied to crosslink and remove proteins and polysaccharides [16]. These silica beads are an eco-friendly, inorganic, non-metallic material, which is commonly known as ultrafine white carbon black [17]. Chlorophyll and other pigments, which are more hydrophobic, tend to dissolve in the PEG solution, while DNA precipitates result in higher purity of the extracted DNA [18]. Additionally, PEG induces changes in the structure of DNA molecules. In solution, DNA can exist in a more extended, coil-like form [19]. When PEG is added, it causes the DNA to collapse into a more compact, globular shape [20]. This conformational change occurs because PEG creates a crowding effect in the solution, which reduces the solubility of DNA and encourages the DNA molecules to aggregate and condense. This property is useful in molecular biology for processes like DNA precipitation and purification, as it helps in separating DNA from other components [20]. Adjusting the concentration of PEG allows for the precipitation of DNA fragments of different sizes. Lower concentrations of PEG are used to precipitate HMW DNA, while higher concentrations are needed to precipitate LMW DNA [20].

Epigenetic modifications provide a tool to distinguish between DNA from different species. For example, cytosine methylation (CpG) is frequently found in eukaryotic genomes including plants, mammals, and insects [21,22,23,24], whereas organelle and prokaryotic DNAs lack CpG methylation [25]. Such differences led to the development of an elegant method to separate CpG-methylated (eukaryotic) DNA by leaving the non-CpG-methylated (microbial) DNA in the supernatant [26]. However, chloroplasts DNA and mitochondrial DNA present a challenge for this method because of their lack of CpG methylation sites [27,28].

*Ca.* Phytoplasma sacchari is an obligate bacterial sugarcane parasite that is difficult to isolate and culture in laboratory conditions. As a result, its DNA must be extracted along with the host plant’s DNA, making selective enrichment of the pathogen’s genetic material particularly challenging [29]. In a prior study, the sequencing data retrieved of the periwinkle leaf yellowing (PLY) phytoplasma genome was only 0.17% of the total Illumina sequencing reads, highlighting the difficult in obtaining sufficient microbial DNA for analysis [30]. Recently, complete genomes of phytoplasma from various plant origins have been reported to be about 474–960 kb including *Ca.* Phytoplasma asteris RP166, SW86, *Ca.* Phytoplasma Australasia, *Ca* Phytoplasma luffae, *Ca.* Phytoplasma phoenicium, *Ca.* Phytoplasma rubi, *Ca.* Phytoplasma sacchari SGGS and SCWL1, *Ca.* Phytoplasma trifolii, *Ca.* Phytoplasma wheat blue dwarf, and *Ca.* Phytoplasma ziziphi [5,7,31,32,33,34,35,36,37,38,39,40]. To retrieve such sparse genome data from the massive nuclear and chloroplast genome content of the plant hosts is undoubtedly an intriguing task.

At present, two genomes of phytoplasma from sugarcane have been reported. The first draft genome sequence of *Ca*. Phytoplasma sacchari SCGS was obtained using a NEBNext microbiome enrichment kit for enrichment and sequenced on both Illumina HiSeq and Oxford Nanopore Technology (ONT) platforms consisting of 29 scaffolds corresponding to 505,173 bp, including a plasmid of 2976 bp, with 95.43% completeness and a coverage depth of 333.98-fold [7]. The complete genome of *Ca.* Phytoplasma sacchari SCWL1 was enriched using a filter-based DNA enrichment method, and 150 bp paired-end sequencing reads were obtained using the Illumina NovaSeq 6000 platform coupled with ONT PromethION sequencing, resulting in 538,951 bp of genomic DNA and 2976 bp of plasmid DNA with 100% completeness and a sequencing depth of 733.85-fold [5]. In this study, the *Ca*. Phytoplasma SCWL whole genome sequence had more contigs and a larger genome size than that of SCGS, but it was smaller than that of SCWL1 with no plasmid detected yet similar completeness to *Ca*. Phytoplasma sacchari SCGS. Notably, our study used only 100 mg of sugarcane leaves and generated sequencing data solely from Illumina NextSeq 500, proving to be cost-effective while covering most of this phytoplasma genome. Although the *Ca*. Phytoplasma sacchari SCWL1 genome achieved 100% completeness with high coverage, it required a larger amount of starting material (5 g) and employed an exclusive combination of Illumina NovaSeq and ONT platforms. Combining size-selective DNA precipitation with other microbial DNA enrichment protocols, such as selective PEG precipitation and differential filtration approaches [5,41], could enhance genome recovery. Despite challenges, such as repetitive sequences, that hinder high-resolution bacterial genome sequencing [42], detecting 98% of the phytoplasma genome suggests that this method is suitable for analyzing genome alterations, including mutations, that may influence pathogenicity. The robustness of our approach demonstrates its effectiveness in assembling a high-quality phytoplasma genome with fewer resources compared to methods requiring more advanced or multiple sequencing platforms.

In addition to the phytoplasma genome, this method has proven the quality of endophytic microbiome data presenting in the SCWL leaves, which are comparable to several reports of endophytic microbes residing within stems, leaves, and roots of sugarcane including Actinobacteria, *Bacillus*, *Burkholderia*, Enterobacteriaceae, and *Pantoea* [43,44,45,46,47].

## 4. Conclusions

In this study, a modified DNA isolation protocol was employed for the isolation of phytoplasma DNA from SCWL sugarcane leaf samples that can be subjected to size-selective DNA precipitation by PEG/NaCl. This alternative DNA isolation protocol employs GuHCl/Silica and avoids the use of chloroform. The size-selective precipitation using PEG/NaCl effectively separates HMW and LMW DNA, enriching microbial DNA in the LMW fraction. With or without the NEBNext Microbiome DNA enrichment kit, shotgun sequencing of the LMW fraction significantly covered almost the complete assembly of the phytoplasma genome regardless of 75 or 150 bp paired-end sequencing data. Thus, this method proves to be efficient, cost-effective, and practicable to generate a high-quality phytoplasma genome compared to other techniques that require more advance sequencing platforms, thus offering a valuable tool for genomic studies with fewer resources. This low-cost method not only enhances the recovery of an unculturable phytoplasma genome from the sugarcane host DNA but also provides a robust framework for studying plant pathogens in other complex plant models.

## 5. Materials and Methods

### 5.1. Sugarcane Samples

Leaves of SCWL cane were collected from the sugarcane plantation of MitrPhol Sugarcane Research Center Co., Ltd., Kalasin province, Thailand (16°42′56.0″ N 103°22′34.2″ E) in December 2018. The leaves were washed with tap water and kept in RNA store reagent (Tiangen, Beijin, China) at −80 °C. The leaves were previously used for a transcriptomic study [48] and kept for long-term storage of 4 years.

### 5.2. DNA Isolation by CTAB Method

The sugarcane leaves were rinsed with sterile distilled water 5 times, cut into small pieces, and finely ground using a mortar and pestle in liquid nitrogen until powdered. Then, 100 mg of the ground sample was transferred in a 2 mL Eppendorf tube containing 1000 µL of CTAB) buffer [100 mM Tris pH 8.0, 20 mM EDTA pH 8.0, 1.4 M NaCl, 4% (*w*/*v*) CTAB, 1% polyvinylpyrrolidone (PVP) and 10 mM β-mercaptoethanol, [49]]. The tube was vortexed and incubated at 60 °C for 1 h, which was followed by treatment with 1 volume of chloroform: isoamyl alcohol (24:1 *v*/*v*) before centrifugation at 15,000 rpm, 4 °C for 10 min. The upper phase was transferred to a new tube, and 0.5 volume of 5 M NaCl was added. DNA was precipitated using 1 volume of isopropanol and pelleted by centrifugation at 15,000 rpm, 4 °C for 10 min. The DNA pellet was washed with 70% ethanol, air-dried, and resuspended in 50 µL TE (10 mM Tris, 1 mM EDTA, pH 7.0).

### 5.3. Preparation of Hydroxylated Silica Beads

All reagents were purchased from Merck (Darmstadt, Germany). Hydroxylated silica nanoparticles were generated by Stöber synthesis [50] by mixing 9.8 mL 29% NH_4_OH, 10.8 mL distilled water, and 73.8 mL 96% EtOH prior to the addition of 5.6 mL tetraethyl orthosilicate (TEOS). The solution was stirred with a magnetic stirrer at 500 rpm for 12 h at room temperature. The silica nanoparticles were then transferred into a 50 mL Falcon tube and collected by centrifugation at 4300 rpm for 10 min. The beads were washed 4 times with 25 mL absolute EtOH and twice with distilled water. Then, the silica beads were dissolved in 1 M citric acid to a concentration of 140 mg beads per mL and dispersed by sonication.

### 5.4. DNA Isolation by Guanidine-HCl-Hydroxylated Silica (GuHCl-Silica) Based Method

First, 100 mg of ground leaf sample was transferred in a 2 mL Eppendorf tube prior to the sequential addition of 450 µL of guanidine-HCl (G2) buffer (800 mM guanidine HCl, 30 mM Tris-HCl pH 8, 30 mM EDTA pH 8.5, 5% tween-20, 0.5% triton X-100), and 50 µL proteinase K (20 mg/mL; iNtRon Biotechnology, Gyeonggi-do, Republic of Korea). The tube was inverted to mix and incubated overnight at 50 °C. Then, another 500 µL of G2 buffer was added and centrifuged at 15,000 rpm, 4 °C for 10 min. The supernatant was transferred to a new tube, and 4 µL of RNaseA (10 mg/mL; Applichem, Barcelona, Spain) was added and incubated at 37 °C for 1 h. To eliminate proteins and polysaccharides [16], 20 µL of hydroxylated silica beads (140 mg/mL) was added, gently inverted by rotation at RT for 10 min, and centrifuged at 15,000 rpm, 4 °C for 5 min. The supernatant was transferred to a new tube, and the above steps were repeated using hydroxylated silica beads. The final supernatant was subjected to the next DNA precipitation protocols.

### 5.5. Size-Selective DNA Precipitation Using Polyethylene Glycol (PEG)

Total DNA precipitation was achieved by adding 0.5 volumes of PEG solution [20% (*w*/*v*) PEG8000 in 2.5 M NaCl] to the final supernatant obtained above. The sample was inverted and stored at 4 °C for 30 min prior to centrifugation at 15,000 rpm, 4 °C for 10 min. After removal of the supernatant, the DNA pellet was washed with 80% EtOH, briefly air-dried, and resuspended in 50 µL TE.

For DNA size selection, 0.5 volumes of PEG solution to the final supernatant were prepared. Firstly, 0.2 volumes of PEG solution were added to the supernatant and stored at 4 °C for 4 h prior to DNA precipitation to enrich the HMW DNA. Lastly, 0.3 volumes of PEG solution were added to the remaining supernatant and stored at 4 °C for 30 min prior to DNA precipitation to enrich the LMW DNA.

### 5.6. Measurement of DNA Quality and Quantity

DNA was analyzed by electrophoresis in a 0.8% agarose gel and stained with SYBR^TM^ safe DNA gel stain (Invitrogen, Waltham, MA, USA). DNA quality was analyzed by Nanodrop (260/280 nm ratio of 1.8). DNA concentration was either analyzed by Nanodrop (260 nm) or quantified using a DeNovix DS-11 FX spectrophotometer/fluorometer (DeNovix, Wilmington, DA, USA).

### 5.7. NEBNext Microbiome DNA Enrichment

Microbial DNA presenting in the fractions of total or HMW or LMW DNAs was further enriched using the NEBNext Microbiome DNA enrichment kit (New England Biolabs, Ipswich, MA, USA) according to the manufacturer’s protocol. In brief, 1 μg of DNA was added to 160 μL of MBD2-Fc-bound paramagnetic beads and mixed by rotation at RT for 15 min. The methylated host DNA (sugarcane) bound to the paramagnetic beads and was separated from the unbound DNA (supernatant) using a magnetic rack. The supernatant containing enriched microbial DNA was transferred to a new tube. The DNA was then precipitated by the addition of 20 µL 3 M NaCl, 2.5 volumes of absolute ethanol, incubation on ice for 1 h, and centrifugation at 13,000 rpm, 4 °C for 30 min. After removal of the supernatant and air-drying, the DNA pellet was resuspended in 15 μL of TE. The host DNA was recovered from MBD2-Fc-paramagnetic beads by adding 150 μL of TE followed by 2 μL of 20 mg/mL proteinase K and incubating at 65 °C for 1 h. The magnetic rack was then used to obtain the host DNA in the supernatant. The DNA was precipitated as described above and resuspended in 15 μL of TE.

### 5.8. Real-Time Quantitative PCR Assay

Real-time qPCR was performed using the ViiA7 Real-Time PCR system (ThemoFisher Scientific, Waltham, MA, USA) with phytoplasma 16S rRNA gene primers (forward 5′-ATTGGGCGTAAAGGGTGCGTAG-3′ and reverse 5′- TCTTCGAATTAAACAACATGATCCA-3′) [51] and plant ITS universal primers (ITS2F: 5′-ATGCGATACTTGGTGTGAAT-3′ and ITS3R: 5′-GACGCTTCTCCAGACTACAAT-3′; [52]). The amplification was carried out in a volume of 20 µL containing 1× iTaq universal SYBR green supermix (Bio-Rad, Hercules, CA, USA), 500 nM of each primer and 1 µL (0.5 ng/µL) DNA template. The thermal cycle conditions consisted of denaturation at 95 °C for 5 min; 40 cycles at 95 °C for 10 s; and 60 °C for 20 s. The relative quantities of phytoplasma 16S rRNA and plant ITS were calculated from cycle threshold (C_T_) values. The data were analyzed with one-way analysis of variance (ANOVA) and Duncan’s test to determine significant differences between groups at *p*-value < 0.05. All statistical analyses were conducted using a R package.

### 5.9. DNA Library Construction and Shotgun Sequencing

The tagmentation reaction was carried out in 15 µL 1× tagmentation buffer [50 mM Tris-HCl pH 8, 50% dimethyl formamide (DMF), 25 mM MgCl_2_] containing 100 ng DNA and 1.5 µL tagmentase (Tn5A; The Proteomic Service, Centro Andaluz de Biología del Desarrollo, Seville, Spain), and then it was incubated at 37 °C for 1 h. The solution was then buffer exchanged using a G-50 sepharose column equilibrated in TE, which resulted in a final volume of 50 µL. Barcoding was carried out in a 20 µL reaction mix containing 10 µL of 2× Nextera PCR mix, 1 µL primer mix (10 µM of Ni5/Ni7 index primers), and 6 ng of fragmented DNA. DNA was amplified by a Bio-Rad T100 Thermal cycler (Bio-Rad, USA) using the following protocol: 98 °C for 30 s, 14 cycles of 98 °C for 10 s, 63 °C for 30 s, and 72 °C for 30 s, and the final cycle of 72 °C for 5 min. The PCR product was size selected between 250 and 600 bps using AMPureXP beads (Beckman Coulter, Brea, CA, USA) according to the manufacturer’s protocol. The quantity and quality of libraries were, respectively, analyzed using a Qubit and Bioanalyzer^®^ 2100 at the Genomics Core Facility (CABIMER, Seville, Spain). DNA libraries were sequenced using Illumina NextSeq500 MID-Output with the sequencing reaction for 20 million reads with either 2 × 75 or 2 × 150 bps read length. For each sample, 4 replicates were analyzed. A summary description of the experimental conditions for each sample is given in Table S1. Raw sequencing data were deposited as SRA under PRJNA1091541 BioProject.

### 5.10. Shotgun Sequencing and Metagenome-Assembled Genomes (MAGs) Analyses

A sample analysis workflow is shown in Figure S2. Cleaned reads were obtained, and duplicate reads were removed by filtering using FASTP (v0.22.0) [53] with default values. Kraken2 taxonomy classification [54] was performed to assign taxonomic labels; data were filtered at a confidence score of 0.1 and at least 10 reads for each sample. The clean reads of all samples were mapped against sugarcane genome of *Saccharum* hybrid cultivar KK3 (ASM2889292) using Bowtie2 (v2.4.4) [55]. The unmapped reads (containing the microbial genome(s) were filtered by SAMTOOLS (v1.13) [56] and mapped against the genome of *Ca*. Phytoplasma sacchari (ASM926810) using Bowtie2. The unmapped reads were also analyzed at the Bacterial and Viral Bioinformatics Resource Center (BV-BRC) Metagenomic Binning and Assembly Service [57] using metaSPAdes [58] for contig assembly, PATRIC [59] for binning, and CheckM [60] for quality metrics to obtain metagenome-assembled genomes (MAGs).

## Figures and Tables

**Table 1:** Metagenome assembled genome (MAG) of *Ca*. phytoplasma sacchari SCWL obtained from the shotgun sequencing data of the enriched total (en-Total), HMW (en-HMW), LMW (en LMW), and non-enriched LMW (LMWWO) fractions.

Fractions	Total Reads/Bases	Coverage	Completeness (%)	MAG Size (% of Full Genome)	Contigs	Mapped Reads (%)
en-Total	437,087/32,781,525	32×	87.82	249,780 (49%)	69	25
en-HMW	174,221/13,066,575	ND	ND	ND	ND	ND
en-LMW_75	641,758/48,131,850	29×	95.83	494,546 (98%)	64	42
en-LMW_150	398,343/59,751,450	64×	91.78	496,806 (98%)	70	39
LMWWO_150	217,519/32,627,850	18×	91.75	371,897 (74%)	104	16

**Table 2:** Comparison of *Ca*. Phytoplasma sacchari SCWL genome data based on enriched methods, sequencing platform, and metagenome assembly pipelines.

Phytoplasma Strain	Microbial DNA Enriched Method	Sequencing Platform	Metagenomic Assembly Pipeline	Genome/ Plasmid (bp)	Coverage (Fold)	Completeness (%)	Contigs	Ref.
*Ca*. Phytoplasma sacchari SCGS	- NEBNext Microbiome DNA enrichment kit	- Illumina HiSeq - ONT	- MEGAHIT v1.1.3 - MetaBAT2 v2.12.1	505,173/2976	333.98×	95.43	29	[7]
*Ca*. Phytoplasma sacchari SCWL1	- Filter-based method	- Illumina NovaSeq 6000 - ONT	- Unicycler v0.5.0	538,951/2976	733.85×	100	1	[5]
*Ca*. Phytoplasma sacchari SCWL	- PEG DNA size selection - NEBNext Microbiome DNA enrichment kit	- Illumina NextSeq 500	- metaSPAdes - PATRIC - CheckM	533,100/-	215×	95.85	45	This study

## Data Availability

DNA sequencing data were deposited as a sequence read archive (SRA) under PRJNA1091541 BioProject.

## Supplementary Materials

The following supporting information can be downloaded at https://www.mdpi.com/article/10.3390/plants13213006/s1. Figure S1: Schematic outline of high-quality sugarcane DNA purification by GuHCl-Silica based method; Figure S2: Workflow for phytoplasma DNA sequence analysis; Table S1: Summary of shotgun sequencing data and read mapping to sugarcane and phytoplasma genomes.
