# Supplementary material for: Phytoplasma DNA Enrichment from Sugarcane White Leaves for Shotgun Sequencing Improvement
Source: Plants (Basel). 2024 Oct 28;13(21):3006. doi: 10.3390/plants13213006 (PMC11548020; doi:10.3390/plants13213006)
Supplement: Supplementary file 1 [file plants-13-03006-s001.zip › plants-3221314-supplementary.pdf]

**Supplementary Materials**

# Phytoplasma DNA Enrichment from Sugarcane White Leaves for Shotgun Sequencing Improvement

Karan Lohmaneeratana[1,2], Gabriel Gutiérrez[3], Arinthip Thamchaipenet[1,4,*] and Ralf Erik Wellinger[2,3,*]

[1] Department of Genetics, Faculty of Science, Kasetsart University, Bangkok 10900, Thailand
[2] Centro Andaluz de Biología Molecular y Medicina Regenerativa, Universidad de Sevilla, 41092 Sevilla, Spain
[3] Departamento de Genética, Universidad de Sevilla, 41012 Sevilla, Spain
[4] Omics Center for Agriculture, Bioresource, Food and Health Kasetsart University (OmiKU), Bangkok 10900, Thailand

* Correspondence: arinthip.t@ku.ac.th (A.T.); wellinger@us.es (R.E.W.)

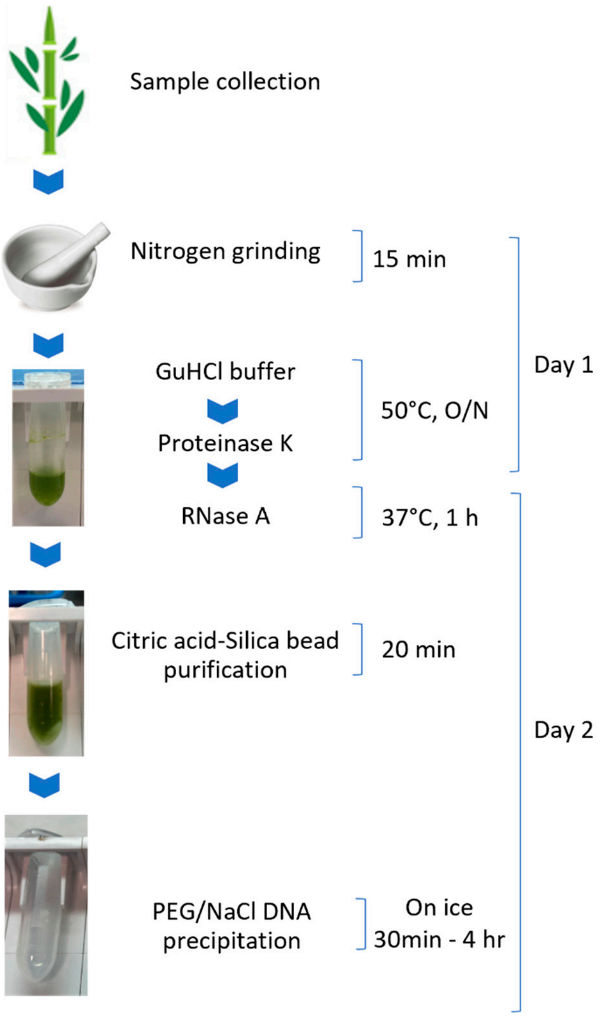


**Figure S1:** Schematic outline of high-quality sugarcane DNA purification by GuHCl-Silica based method.

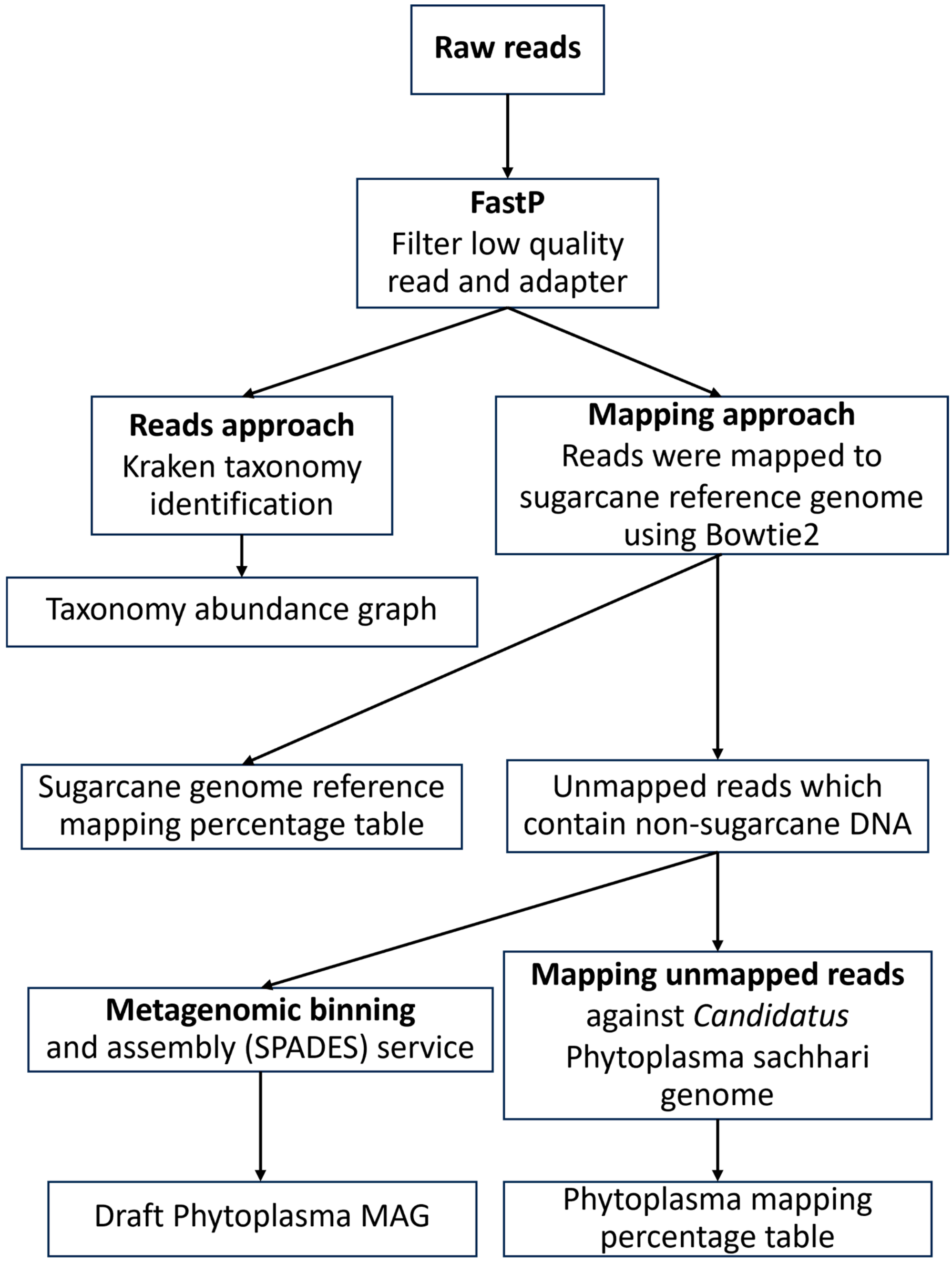


**Figure S2:** Workflow for phytoplasma DNA sequence analysis.

**Table S1.** Summary of shotgun sequencing data and read mapping to sugarcane and phytoplasma genomes.

| Samples (4 replicates each) | NEBNext enrichment | Read length | Number of filtered reads | Mapped reads to sugarcane genome (%) | Mapped reads to phytoplasma genome (%) |
|---|---|---|---|---|---|
| en-TOTAL | yes | 2×75 | 4075571 | 96.26 | 0.67 |
| en-HMW | yes | 2×75 | 4141043 | 98.24 | 0.29 |
| en-LMW_75 | yes | 2×75 | 9772670 | 97.84 | 0.58 |
| LMWWO_75 | no | 2×75 | 10968100 | 98.67 | 0.17 |
| en-LMW_150 | yes | 2×150 | 5585181 | 97.70 | 0.58 |
| LMWWO_150 | no | 2×150 | 5207919 | 98.39 | 0.21 |
